# Advances in the fabrication of graphene transistors on flexible substrates

**DOI:** 10.3762/bjnano.8.50

**Published:** 2017-02-20

**Authors:** Gabriele Fisichella, Stella Lo Verso, Silvestra Di Marco, Vincenzo Vinciguerra, Emanuela Schilirò, Salvatore Di Franco, Raffaella Lo Nigro, Fabrizio Roccaforte, Amaia Zurutuza, Alba Centeno, Sebastiano Ravesi, Filippo Giannazzo

**Affiliations:** 1 CNR-IMM, VIII Strada 5, 95121 Catania, Italy; 2 STMicroelectronics, Stradale Primosole 50, 95121 Catania, Italy; 3 Graphenea, Tolosa Hiribidea 76, Donostia-San Sebastian, Spain

**Keywords:** atomic layer deposition, chemical sensing, field effect transistor, flexible electronics, graphene

## Abstract

Graphene is an ideal candidate for next generation applications as a transparent electrode for electronics on plastic due to its flexibility and the conservation of electrical properties upon deformation. More importantly, its field-effect tunable carrier density, high mobility and saturation velocity make it an appealing choice as a channel material for field-effect transistors (FETs) for several potential applications. As an example, properly designed and scaled graphene FETs (Gr-FETs) can be used for flexible high frequency (RF) electronics or for high sensitivity chemical sensors. Miniaturized and flexible Gr-FET sensors would be highly advantageous for current sensors technology for in vivo and in situ applications. In this paper, we report a wafer-scale processing strategy to fabricate arrays of back-gated Gr-FETs on poly(ethylene naphthalate) (PEN) substrates. These devices present a large-area graphene channel fully exposed to the external environment, in order to be suitable for sensing applications, and the channel conductivity is efficiently modulated by a buried gate contact under a thin Al_2_O_3_ insulating film. In order to be compatible with the use of the PEN substrate, optimized deposition conditions of the Al_2_O_3_ film by plasma-enhanced atomic layer deposition (PE-ALD) at a low temperature (100 °C) have been developed without any relevant degradation of the final dielectric performance.

## Introduction

One of the new challenges in the field of electronics is represented by flexible devices. The evolution of the processing technologies for soft substrates and the discovery of new materials suitable for bending, stretching or conformably shaping [1], such as in the case of many 2D materials [2], paved the way to a huge number of stretchable, foldable or form factor reconfigurable demonstrators. Such devices can be considered for various applications, from consumer devices [3] to biomedical in vivo applications [4–5]. Among all the two-dimensional materials, graphene is one of the most appealing to be used as a flexible, conductive membrane, given its Young’s modulus on the order of TPa and large spring constant (1–5 N/m) [6]. Besides its high charge mobility of up to thousands of cm^2^·V^−1^·s^−1^, even at room temperature for both electron and holes, graphene is characterized by exhibiting only a small variation in electrical performance under mechanical deformation [7–8]. These are essential characteristics for stable and reliable operation.

Thanks to these properties, graphene can be considered for high frequency FETs both on conventional substrates [9] and on flexible platforms [10]. In particular, remarkable cut-off frequency values (≈25 GHz) and robust performance under repeated bending (down to 0.7 mm bending radius) have been reported in graphene FETs (Gr-FETs) even with gradually scaled (≈0.5 µm) channel lengths fabricated on a flexible polyimide substrate and adopting a back-gate configuration and Al_2_O_3_ as a gate dielectric.

In addition, due to the low density of states (DOS) around the Dirac point, the carrier density of graphene is very sensitive to the adsorption of charged/polar species at or near its surface – a peculiarity particularly suitable for chemical/biological sensing. As an example, gas sensors with single molecule sensitivity have been initially demonstrated using high quality, exfoliated graphene from graphite [11]. Considering the scalable graphene production methods, epitaxial graphene grown on silicon carbide has also been demonstrated as an excellent material for sensing [12]. However, for many applications, flexible and disposable sensors are needed. For these applications graphene has to be easily transferred to the target substrate. In this sense, the use of graphene grown by chemical vapor deposition (CVD) on various metals (Ni [7], Cu [13]) and using various precursors [14] represents the most suitable choice.

Among the various device architectures, Gr-FET-based sensors can represent a great combination between a chemical-to-electrical signal converter and an electrical signal amplifier [15]. In particular, the first characteristic can be achieved by using a properly extended channel area able to effectively interact with the chemical target, while the second one can be obtained by maximizing the gate capacitance. For this last reason, from a purely research perspective, the best device configuration is the ion sensing FET (IS-FET) [16] constituted of a graphene channel covered by the target solution and a macroscopic reference electrode immersed in the solution itself as the gate contact. Beside the fabrication simplicity, this configuration exploits electric double layer capacitance at the gate/solution interface and at the channel/solution interface, which can reach tens of µF/cm^2^ (depending on the ions concentration). However, for real applications, especially for a potential totally flexible device, there is a need of a reference electrode that is an external, rigid and macroscopic element, which represents a relevant drawback. The solid IS-FET [17] (where a local back-gate buried under a solid dielectric film replaces the reference electrode) represents a valid alternative for real applications. In this case the thickness and the dielectric constant of the insulating film have crucial importance in order to maintain a reasonably high gate capacitance of the final device. In particular, considering high κ-dielectrics such as HfO_2_ or Al_2_O_3_ with film thickness in the order of 10 nm, the gate capacitance can reach hundreds or even thousands of nF/cm^2^, which is still reasonably high for sensing applications. It is clear that the high quality and the scaled thickness of the dielectric film fabricated below the temperature limit of the plastic support is the key point for the final devices performance.

A low temperature (100 °C) deposition process to obtain a high quality dielectric film is essential in order to be compatible with common plastic substrates, such as poly(ethylene terephthalate) (PET) or poly(ethylene naphthalate) (PEN), which is also the case of our study.

Atomic layer deposition (ALD) represents an optimal method to fabricate a good quality Al_2_O_3_ dielectric film with a tight control on the deposited thickness and a high level of conformal coverage. While the thickness control allows easy fabrication of a tens of nanometer thick dielectric film (resulting in a beneficially high dielectric capacitance), the conformal coverage is essential to contain the potential local degradation of the dielectric performance. Otherwise, this degradation can take over due to the device topography and the high starting roughness of the plastic substrate.

ALD is essentially a low temperature process. It is possible to obtain a high quality Al_2_O_3_ film by depositing using a plasma-enhanced ALD process and exploiting trimethylaluminium (TMA) as the metalorganic chemical precursor and O_2_ as the co-reagent at an optimal growth temperature of 250 °C. Nevertheless, such a temperature is still high in combination with a plastic substrate such as PEN, for which the glass transition temperature is ≈155 °C. Using ALD it is possible to grow at lower temperatures as reported in the case of plastic coating and gas diffusion barrier fabrication [18]. However, with reducing the temperature, the deposition conditions must be optimized in order to ensure the expected layer-by-layer ALD mechanism of the insulating film, instead of a massive CVD growth mechanism, which in turn can result in a degradation of the overall structural and electrical quality of the insulator. This aspect is particularly relevant for the fabrication of a FET where the properties of the gate dielectric (e.g., permittivity, leakage current, critical breakdown field) are crucial for the device operation.

## Results and Discussion

### Low temperature gate dielectric

In our experiments we developed a 100 °C PE-ALD process using a PE ALD LL reactor by SENTECH Instruments GmbH, starting with trimethylaluminium (TMA) as a metal-organic chemical precursor and O_2_ as the oxygen source for the Al_2_O_3_ synthesis. In particular, we explored the resulting properties obtained by modifying the deposition cycle and enlarging the purging periods between the precursor exposure in order to prevent the precursor mixing and a consequent CVD growth mechanism.

**Figure 1:**
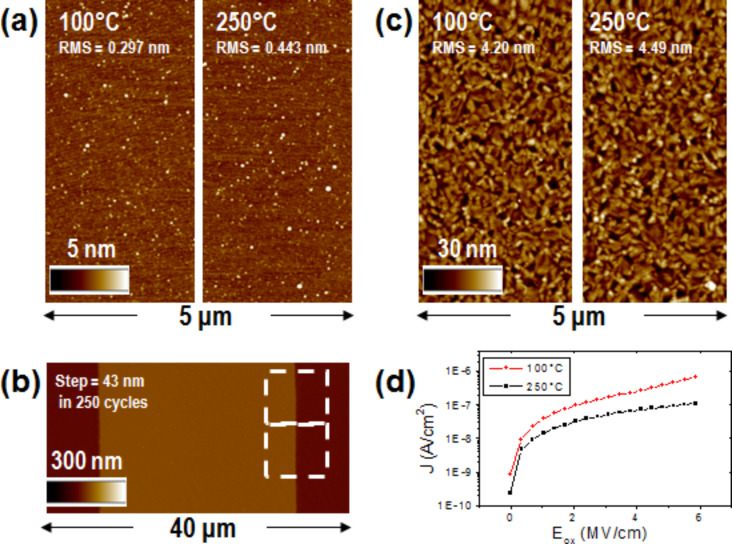
a) Comparison between tapping mode atomic force microscopy (tAFM) morphologies of low temperature (left) and standard temperature (right) dielectric materials deposited on a Si wafer; b) AFM local step height of the lift-off patterned low temperature dielectric; c) comparison between tAFM morphologies of low temperature (left) and standard temperature (right) dielectric material deposited on an Al coated Si wafer; and d) current density leakage through low temperature and standard temperature dielectric materials.

The developed low temperature (LT) and prolonged cycle process was analyzed on a silicon (100) reference wafer and compared to a standard temperature (ST) PE-ALD growth process. The morphology collected by tapping mode atomic force microscopy (tAFM) for the LT growth (Figure 1a left) is comparable to the morphology obtained by the ST growth (Figure 1a right). The dielectric thickness and the consequent growth per cycle was determined by spectroscopic ellipsometry measurements performed on an array of several positions on a wafer scale. A thickness of 28.9 ± 0.5 nm after 250 ALD cycles (0.12 nm/cycle growth rate) was revealed by the ST process, whereas after the same number of cycles, the LT process revealed a thickness of 40.9 ± 0.5 nm (0.16 nm/cycle growth rate).

The LT process thickness was also confirmed (43 ± 1 nm) by the local step height measure reported in Figure 1b for a patterned Al_2_O_3._ The dielectric patterning was obtained by depositing the oxide on a silicon wafer masked by a negative profile resist and then removing the resist by an ultrasonic hot chemical bath. This approach, namely the lift-off method, allows a structured layer to be defined without direct chemical/physical etching of the patterned layer. This completely avoids incomplete or over etching, resulting in a step profile which corresponds to the patterned layer thickness. It should be noted that this method is allowed only for the LT process if a standard resist for lift-off process is considered [19]. The 250 °C used in the ST process would totally degrade the resist.

Both LT and ST processes were repeated on an aluminum-coated silicon wafer, where a number of cycles was needed to produce a ≈30 nm dielectric thickness. The Al-coated Si wafer was prepared by depositing a 200 nm thick Al film by RF reactive sputtering with a surface roughness (measured by AFM, image not reported) of about 4.3 nm. The RMS roughness values obtained for both the LT Al_2_O_3_ (Figure 1c, left) and the ST Al_2_O_3_ (Figure 1c, right) were 4.20 nm and 4.49 nm, respectively, that is, very similar to the starting Al surface roughness.

The Al/Al_2_O_3_ stack was exploited for a mercury probe measurement of the dielectric capacitance. In particular, a metal–insulator–metal capacitor (MIM) is defined by the Al/Al_2_O_3_ stack from one side and from the reversibly contacted mercury probe on the other side. A wafer-scale matrix of sampled positions were collected on both the ST and the LT dielectric layers. Dielectric constants of 7.94 ± 0.05 and 7.91 ± 0.05 where extracted for ST and LT dielectrics, respectively, confirming a similar dielectric quality of the two films and an extremely good homogeneity of the result on the wafer scale (standard deviation below 1%). As reported in Figure 1d the leakage current through the dielectric was also extracted. In both cases, a negligible current on the order of hundreds of nA/cm^2^ (see Table 1) was collected for electric fields up to 6 MV/cm, which is a reasonable operating range. It is possible to conclude that the LT film obtained by a proper modification of the deposition parameters reveals a dielectric quality close to that obtained by a ST film and is good enough to be exploited as a dielectric material for FETs on plastic.

**Table 1:** Comparison between the low temperature and standard temperature process properties extracted during the material characterization on the standard Si substrates.

	Low temperature (100 °C)	Standard temperature (250 °C)

Growth per Cycle:		
* Ellipsometry	0.16 nm/cycle	0.12 nm/cycle
* Local step height	0.168 nm/cycle	–
Relative permittivity	7.91	7.95
Leakage (6 MV/cm)	6.6 × 10^−7^ A/cm^2^	1.1 × 10^−7^ A/cm^2^
Roughness (RMS):		
* On Si wafer	0.297 nm	0.443 nm
* On sputtered Al	4.20 nm	4.49 nm

### Fabrication and electrical characterization of Gr-FETs on flexible substrates

We fabricated several arrays of independently back-gated Gr-FETs by adopting a specifically optimized process flow. In particular, we considered large area devices with channel widths and lengths on the order of ≈100 µm, suitable for solution sensing applications. This channel size poses a challenge considering that the larger the channel dimension, the higher the effect can be on the device performance due to the material defects (e.g., graphene cracks and grain boundaries, surface asperities, dielectric inhomogeneity).

We started with a thermally flattened PEN Teonex film purchased from Dupont Teiji Films. PEN is an analogous material to poly(ethylene terephthalate) (PET), with superior physical and thermal stability (up to 155 °C), which are advantageous properties for flexible technology.

The polymer film was reversibly bonded on a Si wafer by mechanical lamination, adopting a double face thermal release tape (Nitto Denko, 150 °C thermal release) and shaped by cutting along the wafer edges. This is essential for proper manageability of the substrate during fabrication and testing.

**Figure 2:**
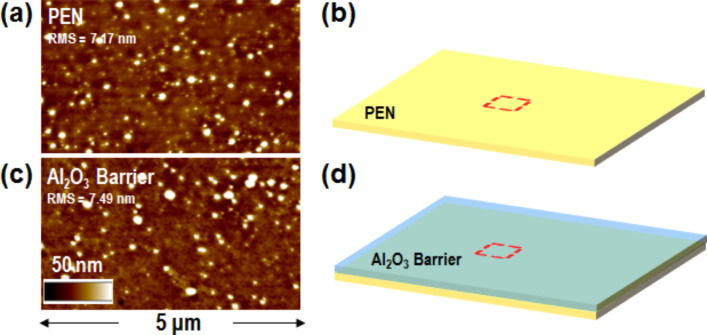
a) Tapping mode atomic force microscopy (tAFM) morphology of the PEN surface and b) a schematic representation of the PEN starting substrate. c) tAFM morphology of the PEN-coated surface by a 100 nm protective Al_2_O_3_ layer and d) a schematic representation of the Al_2_O_3_ barrier/PEN substrate.

The polymer film, as reported in Figure 2a, was morphologically characterized by tAFM and a starting RMS of ≈7 nm was found. It is worth noting that even if this is a reasonable roughness for a plastic substrate, it is 1–2 orders of magnitude higher than the RMS values of traditional rigid substrates for electronics, such as silicon dioxide on Si.

The polymer film surface was coated by a 100 nm protective Al_2_O_3_ layer, deposited by DC-pulsed RF reactive sputter, assisted by a cooling system able to maintain the sample below 100 °C. This is a relevant precaution in order to prevent polymer degradation which may occur due to the processing, in particular, where a plasma is involved. The tAFM morphology reported in Figure 2c shows an essentially unchanged morphology of the substrate surface after the protective coating deposition. This is an important indication of the absence of polymer degradation during the integration of the protective barrier. Figure 2d reports the schematic representation of the PEN substrate after the Al_2_O_3_ barrier integration.

**Figure 3:**
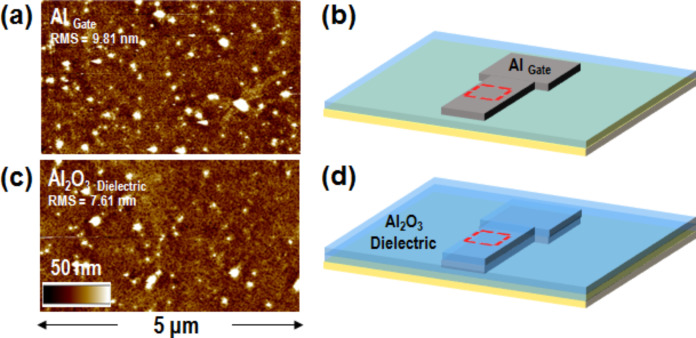
a) Tapping mode atomic force microscopy (tAFM) morphology and b) schematic illustration of the aluminum back-gate pad. c) tAFM morphology and d) related schematic illustration of the Al_2_O_3_ deposited by the low temperature ALD process.

200 nm thick aluminum back gate pads have been fabricated by metal sputtering, considering the same metal deposition process previously exploited on Si during the dielectric layer testing, and patterning by a lift-off approach. The metal morphology is reported in Figure 3a, revealing an RMS of ≈9.8 nm, consistent with the roughness of the underlying substrate. Figure 3b reports the schematic illustration of the patterned Al pads on the plastic substrate.

A ≈30 nm thick Al_2_O_3_ dielectric film was deposited by the previously analyzed LT ALD process. The surface tAFM morphology reported in Figure 3c shows a roughness of ≈7.6 nm, slightly lower than the underlying Al gate contact. The absence of substrate morphological degradation demonstrates that the previously discussed LT process is completely compatible with the final plastic substrate.

Graphene transistor channels were fabricated starting from a single layer graphene film grown by CVD on large area copper foils (provided by Graphenea). The graphene membrane was transferred to a large area (100 mm diameter) of the target substrate by a PMMA-assisted wet transfer procedure and patterned by soft O_2_ plasma etching.

**Figure 4:**
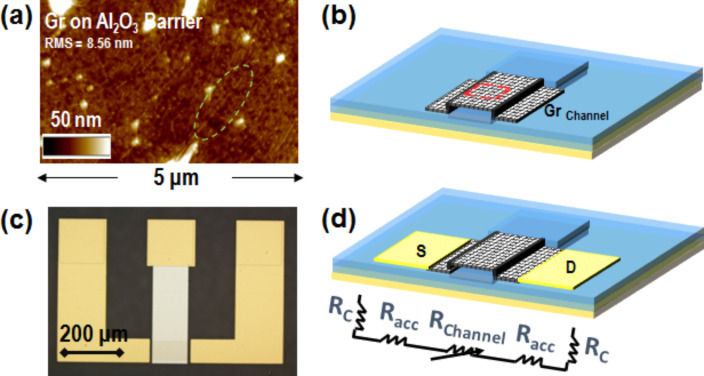
a) Tapping mode atomic force microscopy (tAFM) morphology and b) schematic illustration of the graphene channel. c) Optical microscopy and d) schematic illustration of the final back-gated device with the detail of the involved series resistance contributions from source to drain.

Figure 4a shows the tAFM morphology of graphene. The typical, wrinkled morphology of CVD-synthesized graphene [13] is less evident on PEN substrates compared with smoother substrates. However, it is still possible to identify wrinkles, as indicated by the dashed green circle in Figure 4a. A schematic illustration of the graphene channel fabricated over the FET gate contact is reported in Figure 4b.

After patterning the graphene channel, the source and drain contacts were fabricated by lift-off of a 30/120 nm thick Ni/Au double layer, which partially overlaps the edges of the graphene channel. A complete Gr-FET device is shown in the optical microscopy image in Figure 4c and schematically illustrated in Figure 4d. Furthermore, the resistance contributions which determine the total electrical resistance, *R*_TOT_, between source and drain contacts are also illustrated in Figure 4d. Here, the gate-bias-dependent graphene channel resistance, *R*_ch_(*V*_g_), the source and drain contact resistance, *R*_c_, and the access resistance, *R*_acc_, associated with the ungated graphene access regions between the channel and the source and drain contacts (*L*_acc_ = 20 µm length per access region) are shown.

The total resistance resulting from the series combination of these contributions can be expressed as:

[1]
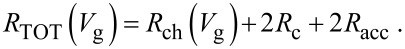


The capacitance of the Al_2_O_3_ dielectric deposited by ALD on the flexible substrate was characterized by metal–insulator–metal (MIM) test devices properly manufactured together with the Gr-FETs, into the same wafer. The resulting gate capacitance per unit area is *C*_g_ = 2.05 × 10^−7^ F/cm^2^. The Al_2_O_3_ film thickness of 29.9 nm is known from the growth rate previously determined on the reference substrate. The resulting dielectric constant is 6.9, which is reasonably high considering the low temperature (100 °C) adopted for the dielectric growth and the high roughness of the substrate. As a way of comparison with silicon dioxide, the resulting equivalent oxide thickness (EOT) is 16.8 nm.

Several arrays of independently biased back-gated Gr-FETs with different channel geometries were fabricated on the wafer scale using the above described process flow. The electrical characterization of more than 50 devices revealed a significant number of early failures. The origin of these failures is out of the scope of this paper and will be the subject of further investigations. Interestingly, working devices showed quite reproducible electrical characteristics.

**Figure 5:**
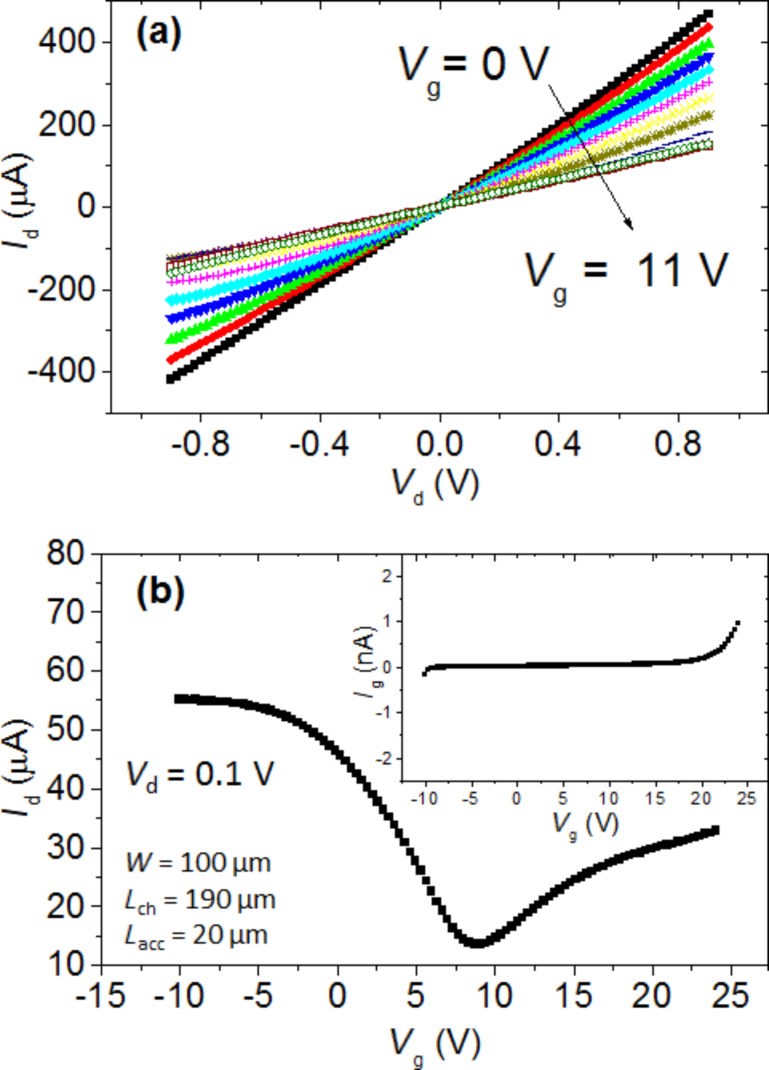
a) *I*_d_–*V*_d_ characteristics at different back gate bias values and b) *I*_d_–*V*_g_ transfer characteristic for a representative Gr-FET with channel width *W* = 100 μm, channel length *L* = 190 μm and access regions length *L*_acc_ = 20 μm. In the insert of b) the leakage current (*I*_g_ vs *V*_g_) collected simultaneously to the transfer characteristic measurement is shown.

Figure 5a reports the output characteristics (drain current vs drain bias, *I*_d_ vs *V*_d_) at incremental values of the back gate bias (*V*_g_ from 0 to 11 V) for a representative Gr-FET device with channel width *W* = 100 µm, channel length *L* = 190 µm (area = *W* × *L* = 19 × 10^3^ µm^2^) and an access region length *L*_acc_ = 20 µm, defined as the distance between source (drain) and the channel region. All the output characteristics exhibit a linear (ohmic) behavior, with a gradually decreasing slope (i.e., an increasing channel resistance) in the considered gate bias range.

Figure 5b shows a representative transfer characteristic of the Gr-FET, i.e., *I*_d_ vs *V*_g_ for a fixed drain bias (*V*_d_ = 0.1 V). It shows the typical ambipolar behavior for a graphene channel with a current minimum at the neutrality (or Dirac) point (*V*_NP_) at 9.0 V. The neutrality point is significantly shifted at high positive bias with respect to the expected ideal value calculated considering the difference between Al and neutral graphene workfunctions (*V*_NP,id_ = *W*_Al_ − *W*_Gr_ ≈ 4.1 − 4.5 = −0.4 eV). This positive shift is a clear indication of graphene p-type doping, as estimated by *p* = *C*_g_(*V*_NP_ − *V*_NP,id_)/*q* ≈ 1.2 × 10^13^ cm^−2^ [20], where *q* is the electron charge. This doping can be ascribed to the effect of the chemical (PMMA) residues which normally persist after the graphene transfer. It is worth noting that due to the constrains imposed by the use of a PEN substrate, it is not possible to perform thermal annealing processes (in vacuum or Ar/H_2_ ambient at temperatures from 300 to 400 °C) or stronger chemical treatments typically used to remove polymeric residues after graphene transfer on standard substrates. Furthermore, a certain doping can be attributed to the electrostatic effect of fixed or trapped charges in the gate oxide when deposited at low temperature.

**Figure 6:**
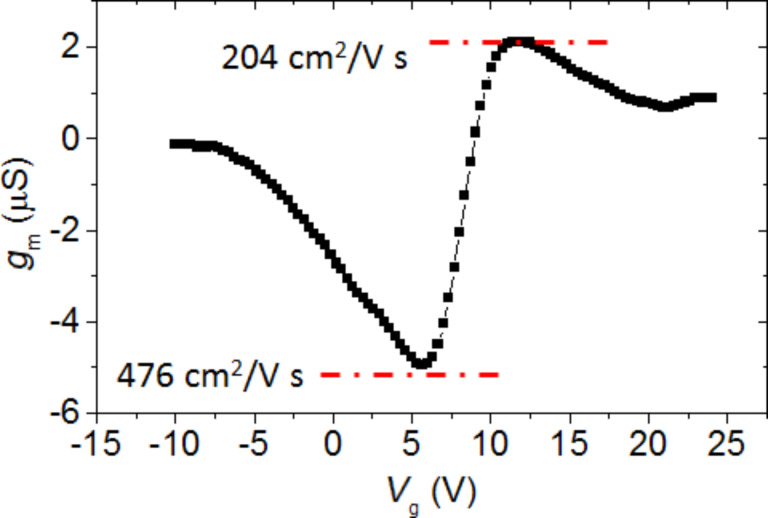
Transfer conductance, *g*_m_, of the Gr-FET, calculated from the *I*_d_ vs *V*_g_ transfer characteristic.

The insert of Figure 5b shows the leakage current through the gate dielectric (*I*_g_ vs *V*_g_) collected simultaneously to the transfer characteristic measurement, showing only a negligible current flow (less than nA) in the whole back gate bias range. The transfer conductance *g*_m_ of the Gr-FET reported in Figure 6 was obtained considering the *I*_d_ vs *V*_g_ characteristic by the formula:

[2]
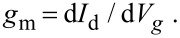


From the peak transfer conductance, we calculated the hole and electron field effect mobility, µ_h_ = 476 cm^2^·V^−1^·s^−1^ and µ_e_ = 204 cm^2^·V^−1^·s^−1^ using the formula:

[3]
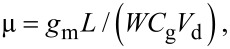


These field effect mobility values are reasonably high, considering the channel area (about 10^4^ µm^2^), the high roughness due to the plastic substrate and the unintentional doping of graphene. In particular, it is expected that the polycrystalline nature of CVD graphene has a direct effect on the current transport in a large area channel. In addition to these natural defects originating from CVD growth, the graphene membrane is subjected to significant strain if transferred to a rough surface while a certain amount of mechanical defects, such as cracks or folding is expected by the transfer procedure itself, especially onto a structured surface. Besides these macroscopic defects, nanoscale scattering mechanisms [21], such as charged impurities scattering and resonant scattering by defects/polymer contaminations, is expected to play a key role in reducing graphene mobility. Finally, it should also be noted that this is a calculation of the extrinsic field effect mobility, where the contribution of the series resistances (such as the contact resistance *R*_c_ and the access resistance *R*_acc_) are still included. A more refined calculation of the graphene field effect mobility would include the elimination of the series resistance contributions in order to extract the intrinsic transfer conductance and the related mobility.

Significant improvements in the transfer conductance and mobility of large area Gr-FETs on flexible substrates will be expected by the use polymeric substrates with optimized lower roughness and by further improvements in the graphene transferring methods, e.g., adopting alternative transfer layers different than common PMMA, leaving a very limited amount of chemical residues on graphene [22].

The field effect modulation of the channel conductivity obtained so far using the thin Al_2_O_3_ back-gate dielectric make the fabricated Gr-FETs interesting as a platform for chemical sensing applications. In this sense, a key step will be proper functionalization of graphene channel to enhance the sensitivity to specific analytes.

## Conclusion

In conclusion, we reported recent advances in the fabrication of Gr-FET with large channel areas (≈10^4^ µm^2^) and local back-gate on flexible PEN substrates. Particular attention was paid to the fabrication of a thin Al_2_O_3_ dielectric film at low temperature. In particular, a low temperature (100 °C) PE-ALD process was optimized and properly tested on a standard substrate (Si) in comparison with a standard PE-ALD deposition. This revealed a material with good morphology, reasonable growth per cycle and comparable dielectric performance. The optimized dielectric material deposition was exploited in order to fabricate back-gated Gr-FETs directly on a PEN substrate, with a gate oxide thickness of 30 nm. Electrical characterization of the Gr-FET devices is reported in order to evaluate key electrical parameters such as the transfer conductance, graphene doping and electron and hole mobility. The fabricated devices will represent the platform for the implementation of solid IS-FETs that can be part of a fully flexible, integrated system for sensing and signal processing.
